# NITS-IQA Database: A New Image Quality Assessment Database

**DOI:** 10.3390/s23042279

**Published:** 2023-02-17

**Authors:** Jayesh Ruikar, Saurabh Chaudhury

**Affiliations:** 1 Department of Electrical Engineering, National Institute of Technology, Silchar 788010, India; 2 Department of Electrical Engineering, Bajaj Institute of Technology, Wardha 442001, India

**Keywords:** image quality assessment, image database, subjective image quality assessment

## Abstract

This paper describes a newly-created image database termed as the NITS-IQA database for image quality assessment (IQA). In spite of recently developed IQA databases, which contain a collection of a huge number of images and type of distortions, there is still a lack of new distortion and use of real natural images taken by the camera. The NITS-IQA database contains total 414 images, including 405 distorted images (nine types of distortion with five levels in each of the distortion type) and nine original images. In this paper, a detailed step by step description of the database development along with the procedure of the subjective test experiment is explained. The subjective test experiment is carried out in order to obtain the individual opinion score of the quality of the images presented before them. The mean opinion score (MOS) is obtained from the individual opinion score. In this paper, the Pearson, Spearman and Kendall rank correlation between a state-of-the-art IQA technique and the MOS are analyzed and presented.

## 1. Introduction

Image quality assessment of digital images is an essential part of image processing applications. Digital images before passing to the end user undergo various processes that introduce discernible distortion in the images. These distortions will deteriorate the image quality. In order to obtain a more enhanced image with a lot less distortion, IQA algorithms are often used. Various IQA algorithms have been proposed over the past and this number is ever-expanding. In order to obtain the universal quality metric that can address all types of distortion, it is essential to develop a new image database for IQA. Addressing a new type of distortion and evaluation of a subjective test grant helps to avoid the hindrances in the IQA database. The development of such an IQA database increases the overall accuracy, improves the performance, reduces the limitation and thus accomplishes results in more robust IQA systems which offer dynamic monitoring and adjustment, optimized algorithms and benchmark systems for image processing [1]. The objective of this study is to understand the aspect of image quality databases and to develop a new image quality database termed as NITS-IQA (National Institute of Technology Silchar—Image Quality Assessment) which will be capable of evaluating and benchmarking image quality algorithms. In recent years, varieties of image quality assessment databases are evolved. The names are enlisted here as CSIQ database [2], IVC database [3], TID2013 database [4,5], LIVE database [6], A57 database [7], MICT database [8], TID2008 database [9], LAR database [10], WIQ database [11] and DRIQ database [12]. All these databases are very famous and widely used, however each of these databases has some limitations which are the driving force forwards the development of NITS-IQA database. The following are considered: (1) Reference image should be all natural. (2) It does not contain an analogue image as one of the reference images. (3) The quality of distorted images is sometimes very poor and unfit to the current digital images. (4) The distortion in all these databases is often similar and created in a similar manner; none of the databases focus on the distortions such as JPEG-XT, pixelate mosaic, contrast change without brightness change, and uniform noise. In this work, we aimed to develop a new image database for quality assessment which is currently to the best of our knowledge the first IQA database in India. 

Development of IQA database consists of three steps: first, capturing the natural images, second, adding various distortions with different levels and lastly, a subjective test experiment. This test experiment set up will be similar to the variation in the number of subjects who are rating the images according to the quality scale provided. The number of test images, types of distortion and levels in each distortion is varying in the IQA database. The success of the IQA database is thus directly related to natural images and distortion that are generally occurring in the images during the various stages of image processing and transmission. In order to proceed further, it is necessary to survey all existing databases to investigate their advantages and disadvantages. Along with the survey, we will propose the process of reference images selection and evaluation. Then, we explore different distortions used in developing the new image quality database.

## 2. Literature Review

Here, we will briefly survey the existing image database available freely for the IQA research community. As they differ by the number of the original images, number of distorted images, number of distortions, level in the distortions, the color of the image etc., it is difficult to classify them. Some of the databases contain the gray images while some others contain color images. In this section, IQA databases which are freely available are discussed here.

The MICT database [8] is a color image database developed by the University of Toyama, Japan in 2008. It consists of 14 reference images in ‘bmp’ format, 168 distorted images and two distortion types, and its subjective assessment is evaluated by the 14 observers. The MOS is calculated from the individual quality score. The quality scale shown to the human observer for the rating ranges from 1 to 5. 

The IVC database [3], is a gray image database developed at the University of Nantes, France in 2005. It consists of 10 reference images in ‘bmp’ format, 185 distorted images, four distortion types and its subjective assessment is evaluated by 15 observers. The MOS is calculated from the individual quality score. The quality scale shown to the human observer for the rating ranges from 1 to 5. 

The LIVE database [6] is a color image database developed by the University of Texas, Austin in 2006. It consists of 29 reference images in ‘bmp’ format, 779 distorted images and five distortion types, and its subjective assessment is evaluated by 161 observers. The differential mean opinion score (DMOS) is also calculated from the individual quality score. The quality scale shown to the human observer for the rating ranges from 1 to 100. 

The A57 database [7] is a grayscale image database developed by the Image Coding and Analysis Lab, Oklahoma State University in 2007. It consists of three reference images in ‘bmp’ format, 54 distorted images and six distortion types, and its subjective assessment is evaluated by the seven observers. The DMOS is then calculated from the individual quality score. The quality scale shown to the human observer for the rating ranges from 0 to 1. 

The TID2008 database [9] is a color image database developed by the Tampere University of Technology, Finland in 2008. It consists of 25 reference images in ‘bmp’ format, 1700 distorted images and 17 distortion types, and its subjective assessment is evaluated by the 838 observers. The MOS is calculated from the individual quality score. The quality scale shown to the human observer for the rating ranges from 0 to 9. 

The TID2013 database [4,5] is a color image database developed by the Tampere University of Technology, Finland in 2013. It consists of 25 reference images in ‘bmp’ format, 3000 distorted images and 24 distortion types, and its subjective assessment is evaluated by 985 observers. The MOS is then calculated from the individual quality score. The quality scale shown to the human observer for the rating ranges from 0 to 9. 

The WIQ database [11] is gray image database developed by the Communications and Computer Systems Laboratory, Blekinge Institute of Technology, Sweden in 2009. It consists of seven reference images in ‘bmp’ format, 80 distorted images and five distortion types, and its subjective assessment is evaluated by 30 observers. The MOS is then calculated from the individual quality score. The quality scale shown to the human observer for the rating ranges from 0 to 100. 

The CSIQ database [2] is a color image database developed by the Oklahoma State University, USA in 2009. It consists of 30 reference images in ‘png’ format, 866 distorted images and six distortion types, and its subjective assessment is evaluated by 35 observers. The DMOS is calculated from the individual quality score. The quality scale shown to the human observer for rating ranges from 0 to 1. 

The LAR database [10] is again a color image database developed by the University of Nantes, France in 2009. It consists of eight reference images in ‘bmp’ and ‘ppm’ format, 120 distorted images and three distortion types, and its subjective assessment is evaluated by 20 observers. The MOS is then calculated from the individual quality score. The quality scale shown to the human observer for the rating ranges from 1 to 5.

The KADID-10k database [13] is an artificial distorted IQA database developed by the University of Konstanz, Germany in 2019. It consists of 81 reference images in ’png’ format, 10125 distorted images and 25 distortion types, and these images were subjectively evaluated by 2209 subjects. Based on the evaluation the DMOS is calculated. The quality scale used in [13] ranges from 1 to 5.

The KonIQ-10k database [14] is a color image database developed by the University of Konstanz, Germany in 2018. It consists of 10073 distorted images. The images are in ‘jpg’ format. The number of subjects involved in the subjective evaluation are 1467. The MOS is calculated on the subjective judgement and it is in the range from 1 to 5.

ChallengeDB [15] is a color image database developed by University of Texas, Austin in 2017. It consists of 1162 distorted images with ‘jpg’ and ‘bmp’ format. The subjects involved in the study of subjective assessment are 8100. 

The SPAQ [16] image quality database consists of 11125 distorted images in ‘jpg’ format. The database is evaluated by 600 observer and MOS is calculated over the range of 0 to 100.

The informative details of popular image databases are presented in Table 1 and Table 2. Table 1 gives details about the name of the database, its year of development, how many references and distorted images are available, number of observers that are used in the subjective study, distortion type, whether the images are of grayscale or color, the format of the images, whether it is an MOS score or a DMOS score and its range. In Table 2, the details of the types of distortions used in the development of popular IQA database are given.

In addition to this DRIQ database [12] is a color image quality database developed by the Oklahoma State University, USA in the year 2012. DRIQ is an acronym for digitally retouched image quality database. Generally, it is understood that the outcome of many images processed gives better visual quality. This process forms the decision of making a digitally retouched image quality database. To the best of our knowledge, the DRIQ database is the only database which contains the enhanced version of the natural images. The DRIQ database contains 26 original natural images and they are edited in Photoshop software to get the better visual quality as compared to the given original image. Therefore, the total database contains 104 images out of which 26 are original images and 78 images are the digitally retouched. Contrast, sharpness, brightness, color or combinations of these at a different level are used to generate the more enhanced natural image. A total of nine observers are used for the subjective evaluation where DMOS has been calculated. The quality scale for the quality rating ranges from 0 to 9.

Out of these databases, two databases i.e., the TID2013 and DRIQ databases need to be noticed due to the following facts: (a) both are new in the IQA research community, (b) TID 2013 contains a greater number of images than any other database present in the IQA research community and (c) DRIQ is the only database which contains the enhanced version of the original image instead of distorted images.

Some of the IQA database is meant for watermarking applications and is made freely available for watermarked IQA community—the Enrico database [17], IVC Broken Arrows (BA) [18] and IVC Fourier Subband (FSB) [19], developed by the University of Nantes, France. Enrico et al. developed the Enrico database [17] in 2007 with five reference images in BMP format, 100 distorted images and 10 distortion types, and its subjective assessment is carried out by the 16 observers. The IVC Broken Arrows (BA) database [18] developed in 2009 has 10 reference images having ‘pgm’ extension, 120 distorted images and two distortion types, and its subjective assessment is carried out by the 17 observers. The IVC Fourier Subband (FSB) database [19] was designed in 2009 with five reference image, 210 distorted images, five types of distortion and its subjective assessment is carried out by the seven observers. Some of the databases are not made available for the IQA research community, presented in [20,21,22], and are used for a specific purpose only. In [20], authors have created the database of 140 images from 10 original images and it uses encoding techniques for creating distorted images. In [21], authors proposed a method for evaluating the quality of color print images and the image database. The database proposed in [21] consists of 40 images made from 10 original images distorted with four types of distortion. In [22], considering the compression artifact, a database is created having 80 images.

Nowadays, significant improvement is seen in the various algorithms being implemented for identification of natural images as suggested in [23], and the classification of image dataset [24]. Moreover, there is significant research carried out in new technologies such as IIOT, big data, block-chain and cloud computing. In [25], a deep learning and IIOT-based ultrasound scanning system is proposed, where image quality must be maintained to diagnose the fetal syndrome. As the images may have complexity and high dimensions, a parallel random forest algorithm for big data in a spark cloud is developed by [26]. To have a secure storage, in [27], blockchain-based methods are proposed.

## 3. Statistical Observation and Analysis of the Benchmark Database

While dealing with MOS or DMOS, it is possible to use descriptive statistics in the analysis of the database. Descriptive statistics on the MOS score over the entire database helps to understand the MOS properties of the central tendency, variation, symmetry and the peakness and flatness. It can be seen from the summary of the database in Table 1 that the range of MOS or DMOS is of varying nature. For example, MICT and IVC MOS ranges from 1 to 5, whereas LIVE and WIQ MOS/DMOS range from 1 to 100. For the statistical observation, it is important to normalize the MOS/DMOS score to 0–1 by using the equation, $x_{norm}=\frac{x_{i}-x_{\min}}{x_{\max}-x_{\min}}$ where $x_{norm}$ is the normalized value for score $x_{i}$, *i* = 1, 2, 3 … and $x_{\min}$ is the minimum value of MOS and $x_{\max}$ is the maximum value of the MOS score in respective database. A descriptive statistical analysis of over the CSIQ database [2], IVC database [3], TID2013 database [4,5], LIVE database [6], A57 database [7], MICT database [12], TID2008 database [9], LAR database [10], WIQ database [11] and DRIQ database [12] is reported in Table 3.

**Figure 1:**
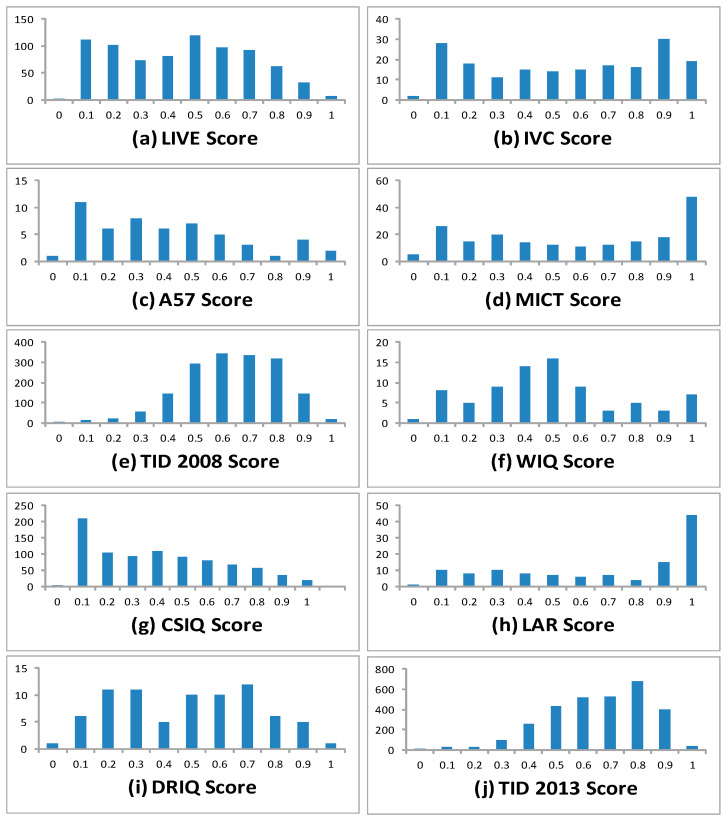
Histogram plot for normalized MOS widely used benchmark database.

The statistics such as mean, median and mode in the table represent the central tendency of the score. Standard deviation and variance represent the spread of the score. From the table, mean and median of the entire database is approximately the same except in the LAR database. Information related to peak or flatness is given by the kurtosis, the positive value represents peaked distribution, while negative is a relatively flat distribution. The degree of asymmetry around its mean is given by the skewness. Positive skewness also called right skewed, represents distribution with an asymmetric tail on the right side, while negative skewness also called left skewed, representing the asymmetric tail on the left side. The last row of the Table 3 shows one of the important measures of dispersion called the coefficient of variation (CV) and is defined as the ratio of the standard deviation to the mean μ: Cv=σμ. As the value of CV increases it means that the data is having a higher variation. The higher the CV, the greater the dispersion in the score. This is suggestive of a good database having huge variation in the subjective assessment score. Furthermore, a histogram of the entire database is evaluated to see the frequency of the normalized MOS over the database. This measure shows the distribution of the MOS score in categorical form (i.e. “Very Poor”, “Poor”, “Fair”, “Good” and “Very Good”) to a scale from 0 to 1 after normalization. The bin range for the normalised MOS is from 0 to 1 with a difference of 0.1 in the horizontal axis. The vertical axis in the histogram plot represents the frequency of occurrence of the normalized MOS at particular bin value. The histogram plot of the normalized subjective score for ten widely-used benchmark databases is shown in Figure 1.

Figure 1e,j represents the histogram plot of the MOS score from TID2008 and TID2013, respectively. It can be seen that there are many images which are lying from 0.5 to 1 range, so generally speaking, these databases are having many images with good quality. Similarly, the Figure 1g shows highest peak at 0.1 indicating the MOS of all the subjects are lying towards the “bad” and “poor” in the categorical scale. From Figure 1h giving the peak at the point 1 in the histogram plot, means again it is having the excellent images in the database. Figure 1a–d,f are showing that these databases contain all variety of images from bad to excellent.

## 4. NITS-IQA Database Overview

Here, we will see the design and development of a new database termed as the NITS-IQA database. The NITS-IQA database is an acronym for the National Institute of Technology Silchar image quality assessment database.

### 4.1. Original Images

**Figure 2:**
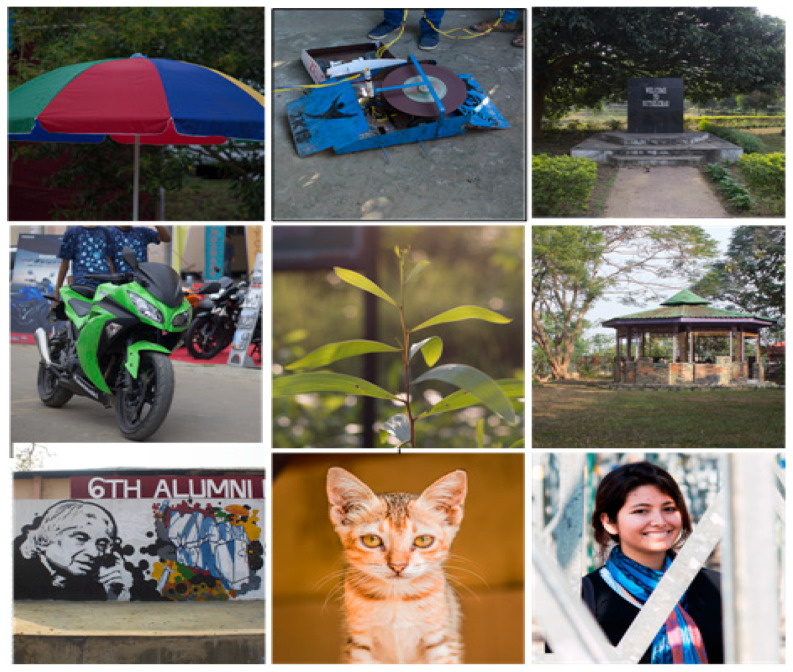
Original images from NITS-IQA database.

In the NITS-IQA database, nine images (Figure 2) are selected after being captured from the campus of NIT Silchar. The images are 512 × 512 pixels, with 72 pixels per inch pixel density. All images are color and in ‘bmp’ format. The size of the images is approximately around 700 kb. The image resolution for all images is 512 × 512 pixels. The reason behind such a selection is based on the resolution of the monitor we have used to conduct the psychophysical experiment. The monitor used is of 1366 × 768 and in order to display two images on the screen side by side we used 512 × 512 pixels images. By considering the area of the image and its sides, we decided the width should be 512 pixels. In addition, some of the image quality metrics processed images block by block and the shape of the block is squared. For this reason, we finally fixed the resolution of the original image as 512 × 512 pixels.

### 4.2. Acquisition Setup

The images present in all the IQA databases referred earlier seem to be quite perfect and mostly chosen from the photographic CD-ROM such as True Color Kodak Images. Hence, we headed to find photos of our own and then go ahead with the analysis. Another reason to use the captured images is to get the optimum and expert opinions over these images in the subjective test. We shot the images with mainly three professional DSLRs, namely Nikon D5100, Canon 60D and Canon 70D. We collected the images in the largest possible forms, i.e., in raw formats of the cameras, they were from—.nef for the Nikon camera and .CR2 for the Canon cameras. The opportunity to gather much larger images from the cameras gave us the opportunity to view the photos from our own angles rather than the set images available under the GPL v2. All the images once taken were then scrutinized (as per the personal judgement) to select the final set of nine images. These images were then transferred onto Photoshop CS6 for further image processing and cropping. On photoshop CS6, the camera raw plug-in was used to directly transfer the data to the main Adobe Photoshop CS6 workspace wherein slight final image enhancements were made before cropping the images into 512 × 512 pixels, with 72 pixels per inch pixel-density in the images. Once this had been carried out, a final archive of all these images was made in ‘.bmp’ format. These images were 24-bit compressed and observed to be around 700 kb in size. The final archive of the original BMP images is used for creating the distorted version of the images for IQA.

### 4.3. Distortions

The NITS-IQA database contains 9 different kind of distortions which are mainly due to compression, noise and blurring artifacts. In the compression artifacts category, JPEG and JPEG2000 [28] compression standards and JPEG-XT [29] standards are used. One can identify the flaws of an image only when one knows the perfection of one in contrast to the non-perfection of the other. Among multiple distortions that can be added to an image, we selected image distortions which were not only plausible but also possible, and happen most of the time to real photographs. The distortions were added to the original BMP images which we have created with the help of Adobe Photoshop CS6.

Table 4 enlists the details of the used distortions such as the name of the distortion, its purpose, when it generally occurred, parameters which are changed while applying distortion and its low value and higher value.

In NITS-IQA database, all the reference images are distorted by these distortions (Table 4) in five levels (intensities) from a low degree of quality degradation to a high degree of quality degradation. The brief introduction of distortions, the arguments of the distortions selection and distortions implementation is given here.

#### 4.3.1. Gaussian Blur

This type of distortion is usually seen when a photographer misses focus. On a range from perfect focus to a complete miss is depicted well in the levels hence induced into the distorted photos created via Adobe Photoshop. Mathematically, it is the convolution of the image with the Gaussian function which acts as low pass filter by reducing the high-frequency component.

#### 4.3.2. Gaussian Noise

It is the statistical noise which has probability density function (PDF) equal to that of the Gaussian distribution. While adding Gaussian noise, more noise is added to mid tones and less noise to shadows. This type of distortion is mostly encountered when the photographer has hiked up the sensitivity (ISO of the camera or) of the screen and still maintain an even exposure across the image. Here, due to the sensitivity hike, the corresponding output gathers a huge amount of noise and as such type of distortions are commonly found. The images distorted with Gaussian noise are constructed in Photoshop.

#### 4.3.3. Uniform Noise

It adds random color noise of equal intensity all over the image. The effect is noticeably more subtle than Gaussian noise. Much like Gaussian noise, this distortion is commonplace due to high ISOs while taking photographs. Images distorted with uniform noise are constructed in Photoshop.

#### 4.3.4. Contrast Change

This is normally the kind of change that a photographer does either on board a camera or on his editing software. This distortion or rather a change in a particular parameter is done to analyze a particular set of contrast changes cause a variation in the perception when applied to different images. It is also created using Photoshop.

#### 4.3.5. Pixelate Mosaic

A similar type of distortion was mostly observed when a photographer sends his/her images though serious compression for easier and data cheap transmission created in Photoshop by changing cell size in the level of 2, 3, 4, 5 and 6.

#### 4.3.6. Motion Blur

As the name itself says, this type of blur is very commonly captured by photographers, sometimes artistic, other times due to the camera/hand shaking during shooting. This is a distortion that greatly affects the quality of output from mostly amateur photographers. It is created in Photoshop with the five levels of 1, 2, 4, 6 and 10.

#### 4.3.7. JPEG Compression

JPEG is an acronym for Joint Photographic Experts Group. This group formed JPEG standards for digital images to decide the codec for image compression and decompression of the image. JPEG uses method of lossy compression for images compression. With the help of Matlab command, JPEG-distorted images are created in the five levels of 12, 20, 35, 50 and 99.

#### 4.3.8. JPEG2000 Compression

JPEG2000 is also an image compression standard and is created by the Joint Photographic Experts Group Committee in 2000. Compared to the original standard JPEG, which is based on discrete cosine transform, the newly designed JPEG2000 is based on wavelet transform. The JPEG2000 compression standard has a significantly better performance than JPEG standard. Images are created using software provided in [28].

#### 4.3.9. JPEG-XT Compression

It is the extension of JPEG known as JPEG-XT (ISO/IEC 18477) that specifies a series of backward compatible extensions to the legacy JPEG standard (ITU Recommendation T.81 | ISO/IEC 10918-1). JPEG-XT develops to eliminate limitation of the JPEG over the higher bit depths (9 to 16 bits), high-dynamic range imaging, lossless compression and representation of alpha channels. In the database, images are created using the free software codec provided in [29].

The complete compilation of distortions and its value at each level that we added to original images to create distorted image is summarize in Table 5. The maximum and minimum range of the distortion and its unit is given in Table 5.

### 4.4. Distorted Images

**Figure 3:**
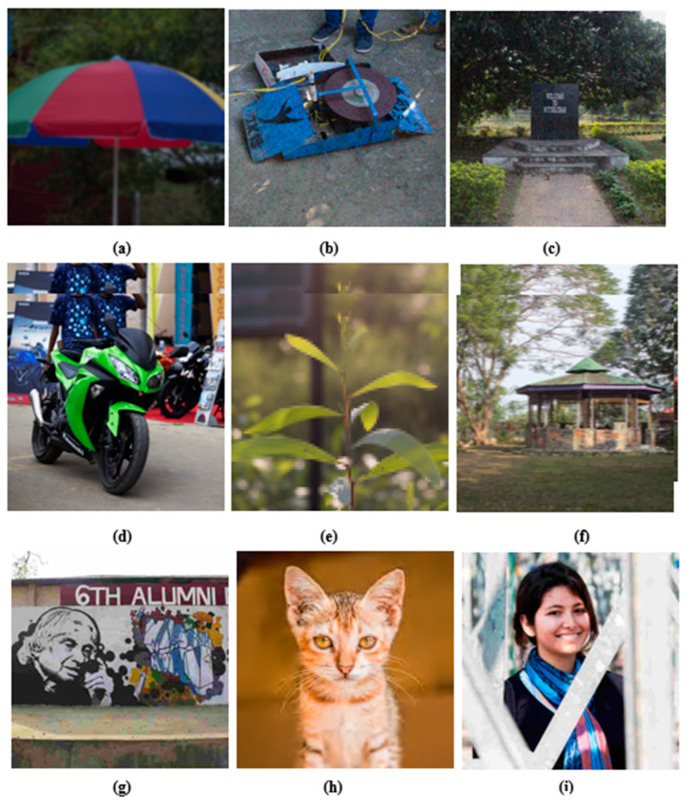
Distorted images sample (**a**) Gaussian blur, (**b**) Gaussian noise (chromatic), (**c**) uniform noise (chromatic), (**d**) contrast change (without brightness change), (**e**) pixelate mosaic, (**f**) motion blur, (**g**) JPEG, (**h**) JPEG2000, (**i**) JPEG-XT.

A total of 405 images are generated by using the 9-reference image disturbed by 9-type distortions with 5-level variations. The level of distortion added to the image is unique and it is same for all the images that have the same level. The names of the images in the database are unique and informative. All the images in the database are in the ‘bmp’ format. Let us consider an image having the name I1D2L4.bmp, I1 represents the 1st original image, D2 represents the 2nd distortion and L4 shows 4th level of variation. Thus, we have images having the name from I1D1L1.bmp, I1D1L2.bmp,.,.,.,, I9D9L4.bmp and I9D9L5.bmp. Figure 3 shows the sample of distorted images with various distortion.

### 4.5. Procedure for Adobe Photoshop CS6

The Adobe Photoshop CS6 gives the possibility to change the type of workspace we work on a particular project and hence for this purpose we used the photography workspace. The images were imported from the camera to the PC in raw formats such as .nef (for Nikons) or. CR2 (for Canons) to Photoshop, and then each image was first cropped in 1 × 1 form using the crop tool in the left tool pick stack.

Once the tool is selected, the drop down is selected for 1 × 1 (Square) in the top toolbar specifications and the required art of the image is selected by dragging the corners of the crop tool in the workspace.

Using this technique once the area is to be cropped when selected, the enter key is pressed on the keyboard to select and crop the image. The image is then cropped and the next step is to bring the pixel density of the images to the standard 72 ppi. For this purpose, the ‘Image Size…’ option is opened from the Image menu option which is present in the menu bar of the window. Then, firstly the textbox next to the ‘Resolution’ Label is set to ‘72′ and ‘Pixels/Inch’ then on in the ‘Pixel Dimensions’ part of the dialogue box the Width and Height-labeled textboxes are set to ‘512′ and ‘Pixels’. After this, ‘Okay’ is pressed to apply these settings. Then, from the ‘File’ menu option ‘Save As…’ option is selected and in the dialogue box that appears, the file name is set in the order of <Image_no.>, e.g., I1, I2, I3… and so on. The format is set to ‘BMP (*.BMP*.RLE, *.DIB)’ and the ‘Use Lower Case Extension’ checkbox is selected and the image is then saved in the archive, hence created. This process would mark the end of the creation of original.bmp format images. The process is repeated for every image.

To generate the images in their respective distorted ways, we generated ‘Actions’. ‘Actions’ is a very powerful feature of Adobe Photoshop in which a specific set of manipulations carried out on an image can be serially saved and then copied onto multiple images in the exact way the manipulations were made in the first image. To make a new action we pressed Alt + F9 where a right tab opened and we henceforth added a new set to the root where there already existed a set called Default. For this case we named the new set as ‘NITS-IQA’. Further, new actions were created by pressing the ‘Create New Actions’ button on the bottom of the Actions pane.

**Figure 4:**
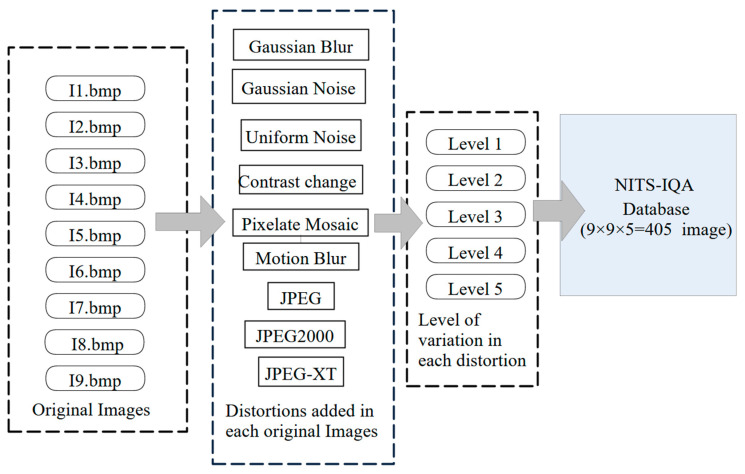
Development stages of NITS-IQA database.

Distortions were created using Photoshop in a similar manner. For example, here we describe the method for adding Gaussian blur. Let us first take into consideration the Gaussian blur distortion. Here the first action was named ‘Guassian_Blur’. By pressing on the ‘Play’ button while selecting the Guassian_Blur action, we initiated the process of recording our manipulations. In a similar way, we added Gaussian noise (chromatic), uniform noise (chromatic), contrast change (without brightness change), pixelate mosaic and motion blur. JPEG-distorted images are developed in Matlab, and JPEG2000 and JPEG-XT are created using the software provided by [28,29], respectively. The basic block diagram to understand the development of the NITS-IQA database is shown in Figure 4.

## 5. Subjective Experiment

In the traditional objective IQA, the quality of the natural images is evaluated by error difference, structural difference and naturalness. It is also found in the literature that human eyes are sufficiently capable to detect the blur present in the image or the edges in the image. Therefore, we can understand human behaviour over the distorted image in the subjective study. After knowing the response from the observer, we can design a metric for quality assessment by employing the HVS model. For the purpose of subjective image quality testing, several international standards are proposed in [30,31,32,33,34,35,36]. The subjective quality assessment is a psychophysical process and is essential to understand and analyze the quality of experience of the observer. In a real situation, unknown and multiple distortions occurred during the various process of the image. Therefore, understanding the subjective responses of observers to numerous perceptual distortions is helpful for improving the objective image quality metric (IQM).

Here, we develop a new database and focus our attention on the psychophysical response to the subjective quality assessment of the NITS-IQA database. This database contains nine images with nine types of distortions with five levels of variations. For participant selection and test surroundings setup, the International Telecommunication Union (ITU) has given some guidelines which are available at ITU-R B.T. 500 [30].

### 5.1. Subjective Test Setup

The subjective experiment setup is carried out in a laboratory environment with normal indoor illumination. All the display monitors used are of CRT Type, 21-inch, having a resolution of 1024 × 768 pixels. All monitors adopted are of the same setting of calibration. The normal distance between the subject and the display is around the 3.5 to 4 H (screen height).

**Figure 5:**
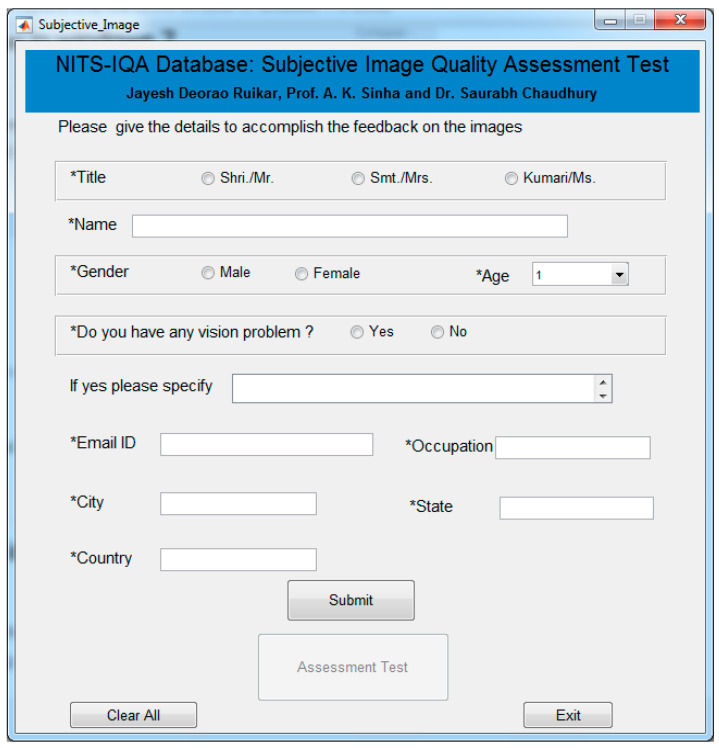
Graphical user interface (GUI)-1.

**Figure 6:**
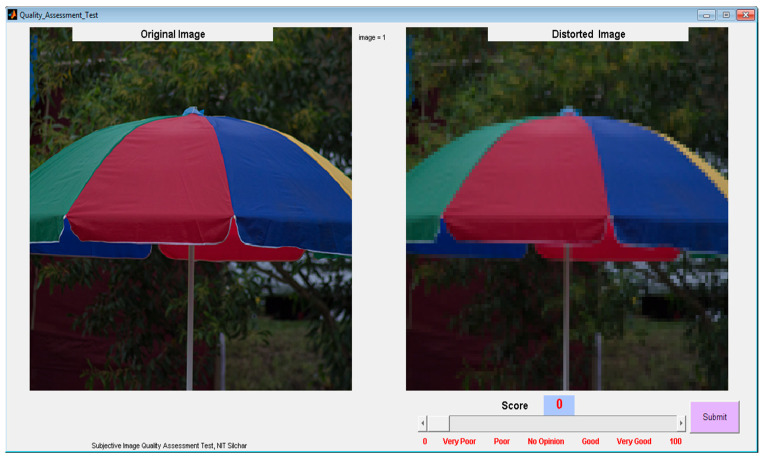
Graphical user interface (GUI)-2.

To carry out our psychophysical subjective test, a Matlab-based graphical user interface (GUI) was created and experiments were arranged. The GUI comprises of two windows that come one after another. The first window shown in Figure 5 collected general details of the participants such as name, gender, occupation, issue related to the eye site and their address. After the collection of general information, users had to click on the submit button. After clicking the submit button, subjective test pushbutton highlighted, which opened the other window as shown in Figure 6. The second window contains two image blocks, a slider, an image score text box, an image number text box and the two pushbuttons (submit and the next image, highlighted one after another). Here, the user can see the original image and randomly selected distorted image placed side by side.

Observers could view the image, regardless the type of distortion and level of distortion used. One slider was provided in the GUI for the observer to choose the quality of the images in 0 to 100 scales. The chosen image score was seen in the text window above the slider to assure the correct judgment. Once the observer had finalized the image score, the observer had to click on the submit button, which signified that the image score had been submitted and the slider automatically reset to its initial position. In order to assess the image score of another image, the observer had to click on the next image button placed in the GUI.

When the observer clicked on the next button, the randomly selected original image and the distorted image was displayed. This procedure was continued until the observer completed the whole experiment. The number of images that were assessed by the observer could be seen in the small text box. With the help of submit and next image push buttons, observers were not allowed to skip any image without judging its score. The slider did not allow users to skip an image without making a judgment. The slider value reset to zero when a new image was displayed. Observers were allowed to take the break in between the subjective test and given refreshments from time to time. All the subjective experiment procedures were same for each observer, and an independent assessment has been carried out to avoid the interference. A quality score from 1 to 100 is used equivalently to specify five grades of images such as, “Very Poor”, “Poor”, “Fair/No Opinion”, “Good” and “Very Good”.

### 5.2. Participant and Training

There is no standard for the number of observers required to conduct the subjective setup. It depends on the availability of the participants. It is suggested in [37] that a minimum of four observers and a maximum of 50 is required while CIE standards suggest that at least 15 are required. The number of participants may vary according to the availability. The basic principle behind the selection depends on the accuracy of the subjective experiment. As the number of participants increases, judgement accuracy also increased. The subjective assessment of the NITS-IQA database was carried out at two locations in several sessions: NIT Silchar and Pune University. The participants were a mixture of expert and non-expert in order to avoid the biased judgement. The faculty members and students from various departments of the National Institute of Technology, Silchar, Assam, India took an active part in the subjective experiment. The students from Pune University, Pune, India also volunteered in the subjective assessment.

The participants consisted of male and female, and most of them were non-expert in the field of image processing. The participants were from three countries such as India, Afghanistan and Bhutan. All the participants were new to study and the literature reveals that these countries are neither involved in the development of the IQA database nor had they carried out a subjective assessment. Therefore, it is interesting to see the responses of these participants in the subjective IQA. This subjective test involved 162 participants who volunteered to participate. None of the participants went through any medical test prior to the subjective test. All the participants were trained before the subjective assessment.

**Figure 7:**
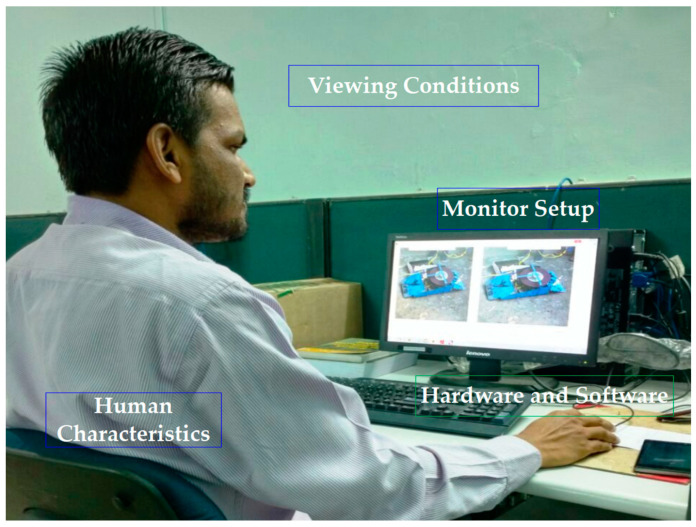
Example of subjective test and its setup.

The participants in the subjective experiments were given the training before starting the test via a dummy presentation. Both the windows were shown and they were asked to fill in the details. The randomness of the program was also described so that there were far fewer chances of getting the same distorted image on the neighboring computer. This subjective experiment was conducted with a minimum of one break while observers were assessing the image quality. Figure 7 showed the example of a participant who was engaged in the subjective test for the NITS-IQA database.

### 5.3. Data Archiving and Exporting

The computer used for the subjective experiments was connected through a local area network (LAN) and contained the necessary programs for the subjective experiment. The results of the subjective experiments were stored in the text file with typical naming conventions. The naming convention included the name of the observer, and the date and time arranged in a specific manner. This reduced the chances of duplicate files with similar name. All the files were exported through the network to a common folder.

### 5.4. Data Processing

**Figure 8:**
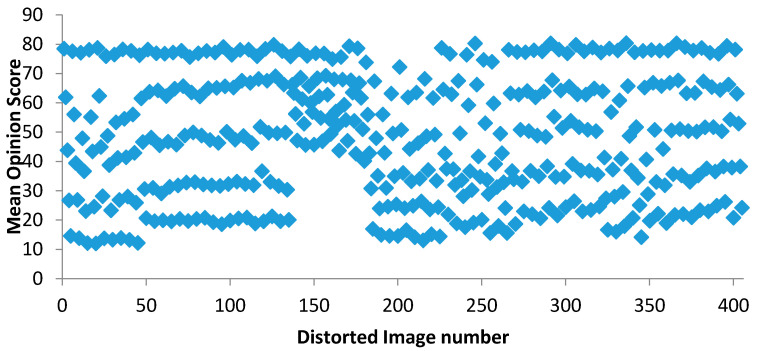
Average of the MOS for reference image.

After the subjective experiments, the results from unfinished and abnormal experimental data were rejected. Altogether, 162 observers had carried out a subjective experiment which gave 65,610 scores across 405 images. The data in all the text files were then collected and checked for the outlier. Here the participants are asked to rate the image quality over the scale of 1 to 100 where 1 represents the very low image while 100 represented very high-quality image. As per the guidelines of the International Telecommunication Union (ITU), the mean opinion score was calculated after removing the outlier present in the score. The mean opinion score is the arithmetic mean calculated over the subjective score. It was calculated as: $MOS=\frac{1}{n}\overset{n}{\underset{i=1}{\sum }}Z_{i}$ where *Z_i_* is the score and n is the number of observers. The average of the MOS for each image was plotted and shown in Figure 8.

## 6. Comparative Analysis of Image Quality Methods

The NITS-IQA database is a natural IQA database comprising of a total 414 images including 405 distorted images (nine types of distortion with five levels in each of the distortion types) and nine original images. These images were taken from the campus of the institute to ensure accurate judgement. The subjective test was carried out in the lab with respect to required environmental conditions. The participants of this subjective experiment were from three countries including India. All participants’ personal information such as name, age, gender, eyesight information and opinions were also stored in a separate file.

The performance evaluation indicator of the proposed NITS-IQA database is based on the correlation coefficient (CC) and the root–mean–square error (RMSE). Here, the correlation between the subjective method of assessment and objective method of assessment was calculated. The subjective method of assessment involved the MOS of the images, whereas the objective method of the assessment involved various metric proposed in the literature for IQA. In this paper, we have used objective image quality metrics (IQM) such as MSE, PSNR, SSIM [38], IFC [39], VIFP [40], VSNR [41], P_HVS_M [42], P_HVS [43], RFSIM [44], FSIM [45], ADM [46], IWSSIM [47], IWMSE [47], IWPSNR [47], SRSIM [48], GSM [49], IGM [50], GMSD [51], UQI [52], MSSIM [53] and WSNR [47] for finding out the correlation coefficient. Here, we used Pearson’s linear correlation coefficient (PLCC), Spearman’s rank order correlation coefficient (SROCC) and Kendall’s rank order correlation coefficient (KROCC) methods to evaluate proposed database over the above stated IQM. In addition to the correlation coefficient, root–mean–square error (RMSE) was also used as a performance indicator of the proposed database.

**Figure 9:**
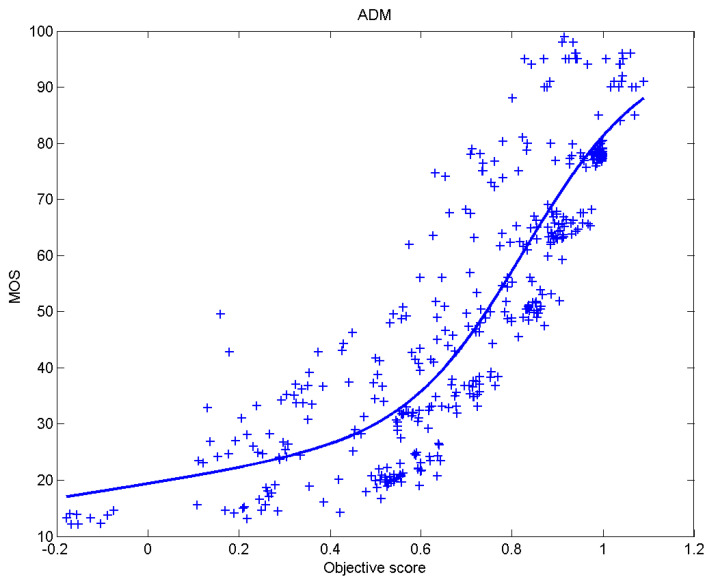
Scatter plots of the subjective scores (MOS) versus ADM.

**Figure 10:**
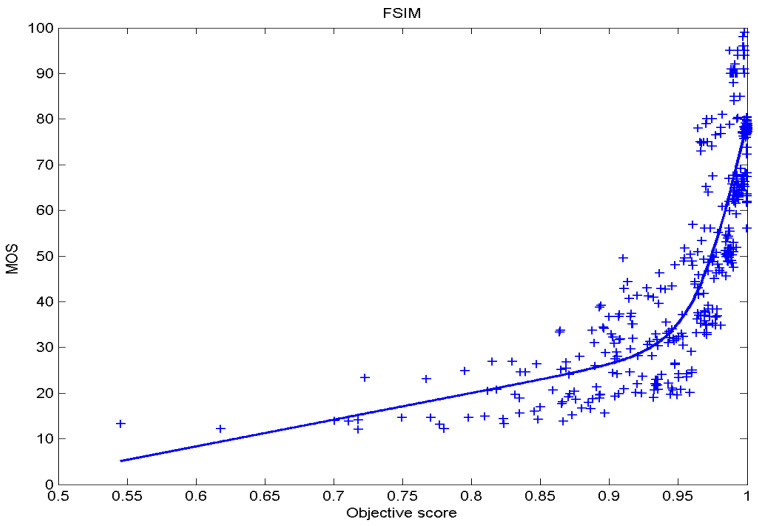
Scatter plots of the subjective scores (MOS) vs. FSIM.

**Figure 11:**
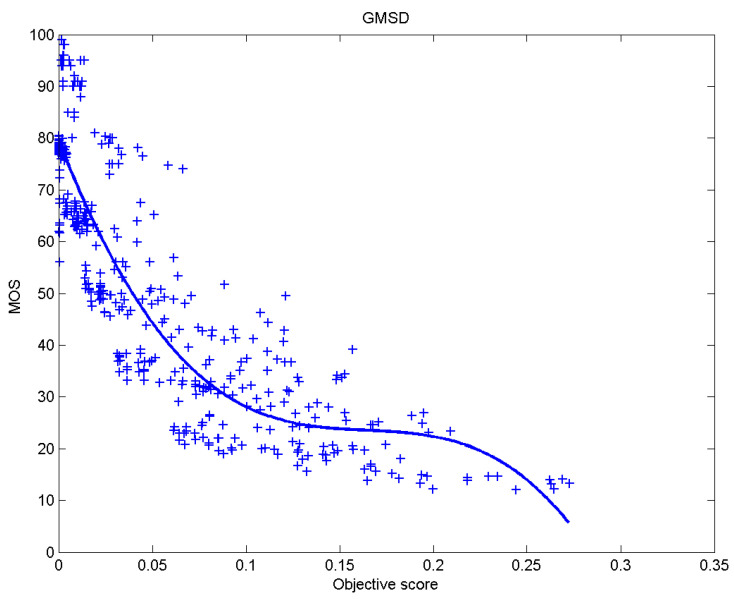
Scatter plots of the subjective scores (MOS) vs. GMSD.

**Figure 12:**
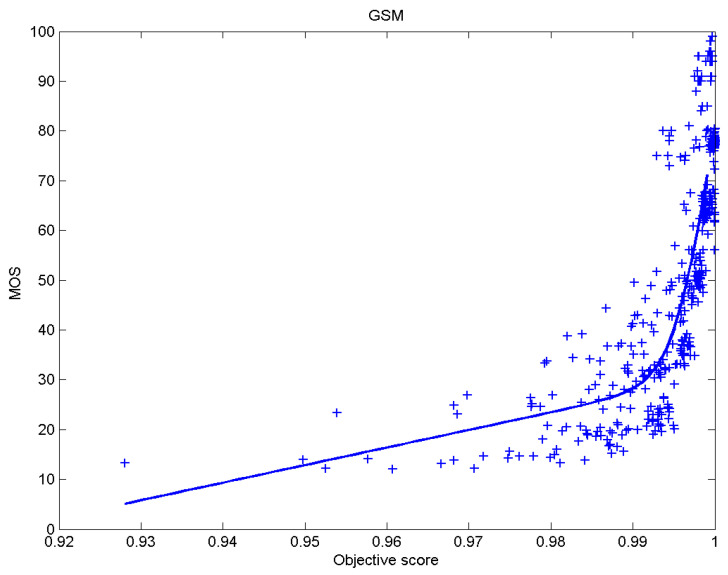
Scatter plots of the subjective scores (MOS) vs. GSM.

**Figure 13:**
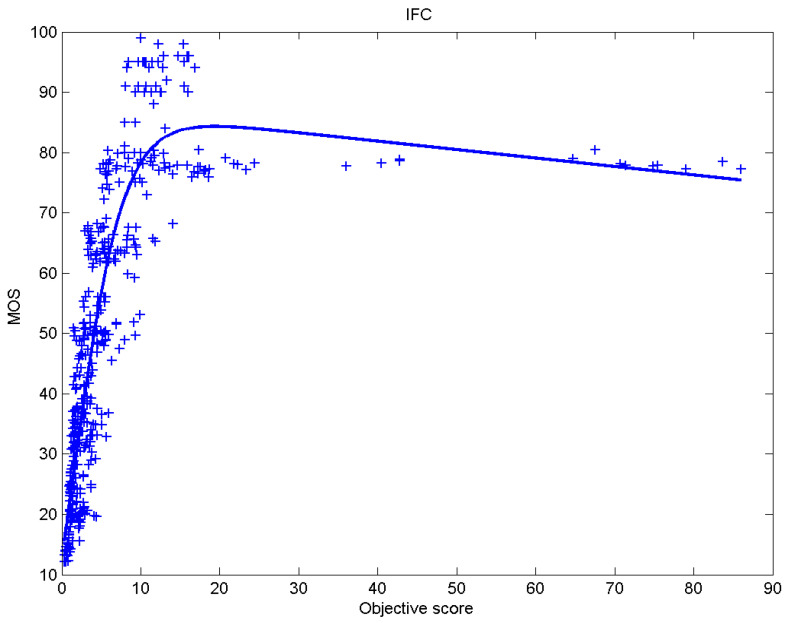
Scatter plots of the subjective scores (MOS) vs. IFC.

**Figure 14:**
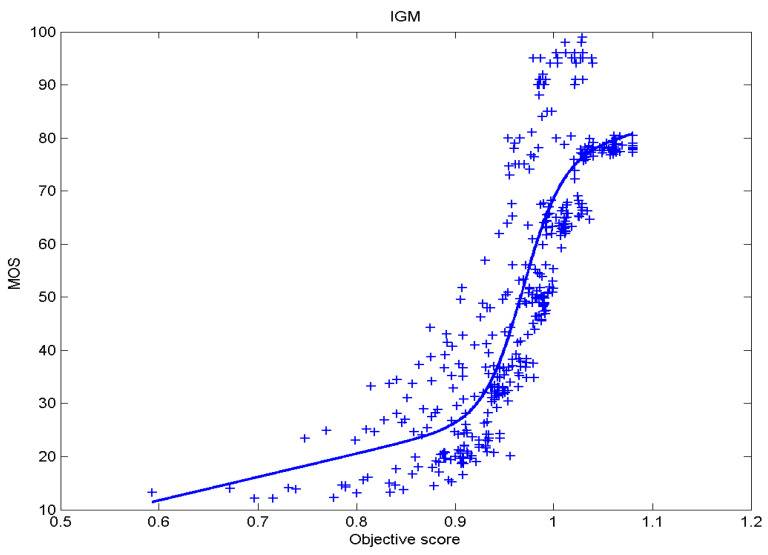
Scatter plots of the subjective scores (MOS) vs. IGM.

**Figure 15:**
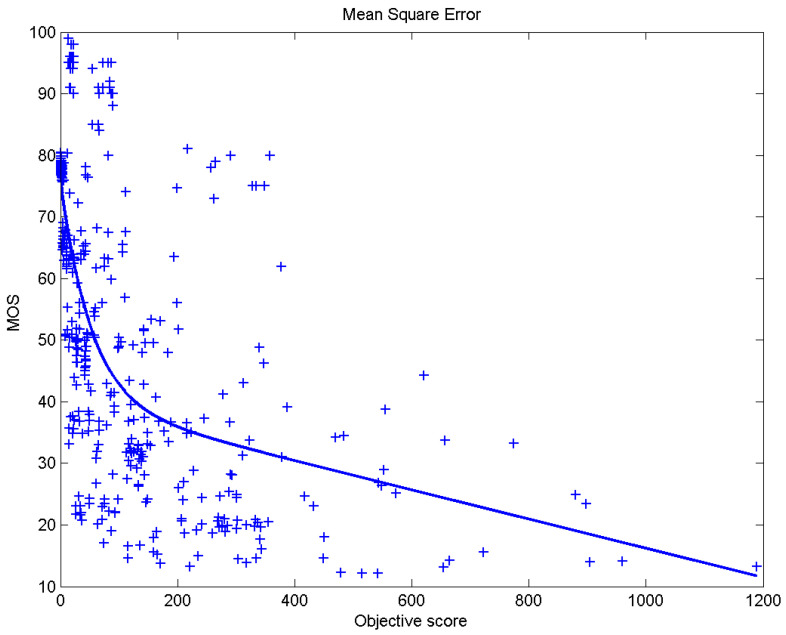
Scatter plots of the subjective scores (MOS) vs. MSE.

**Figure 16:**
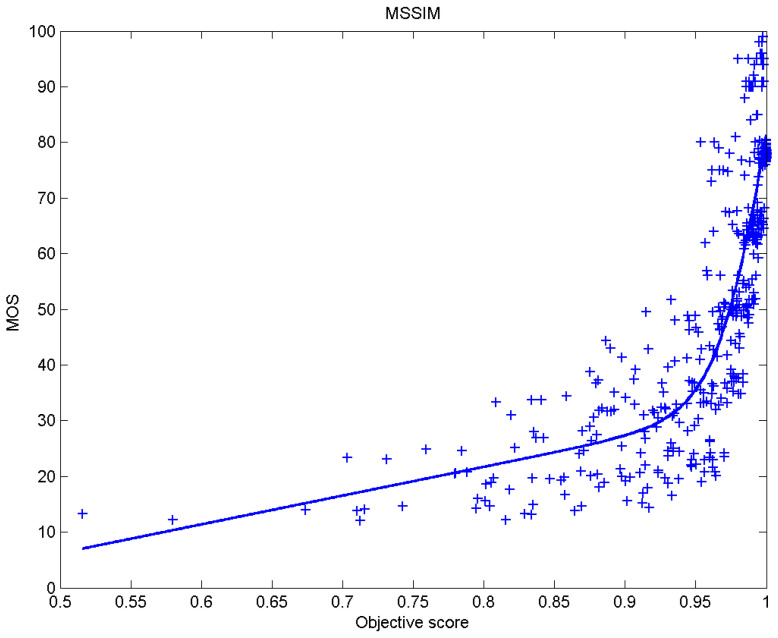
Scatter plots of the subjective scores (MOS) vs. MSSIM.

**Figure 17:**
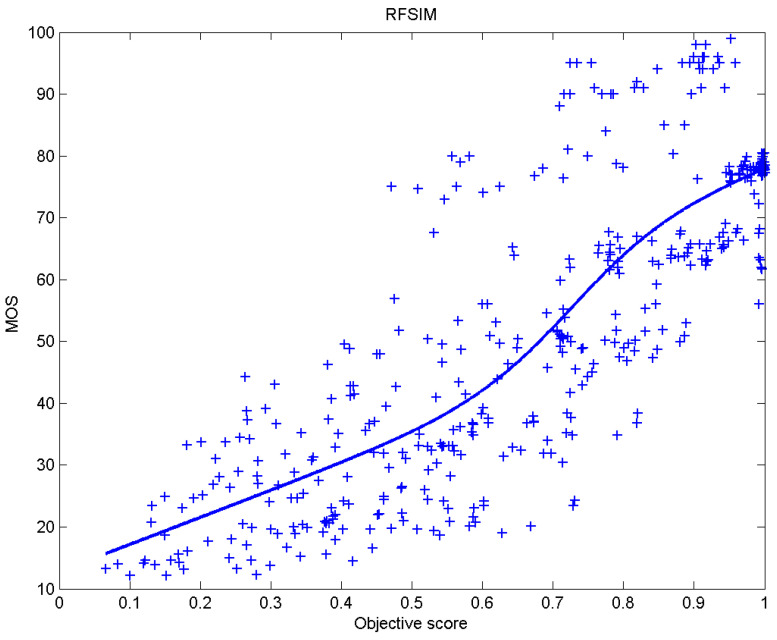
Scatter plots of the subjective scores (MOS) vs. RFSIM.

**Figure 18:**
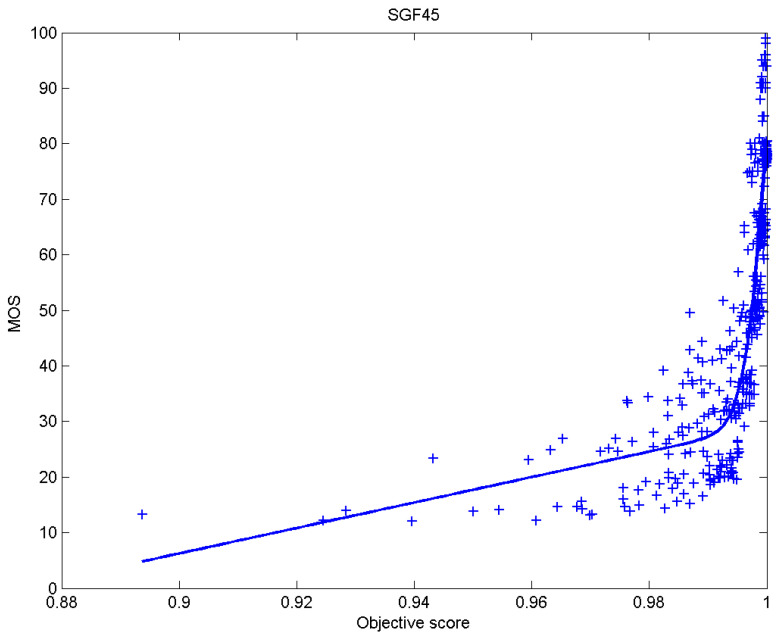
Scatter plots of the subjective scores (MOS) vs. SGF method.

**Figure 19:**
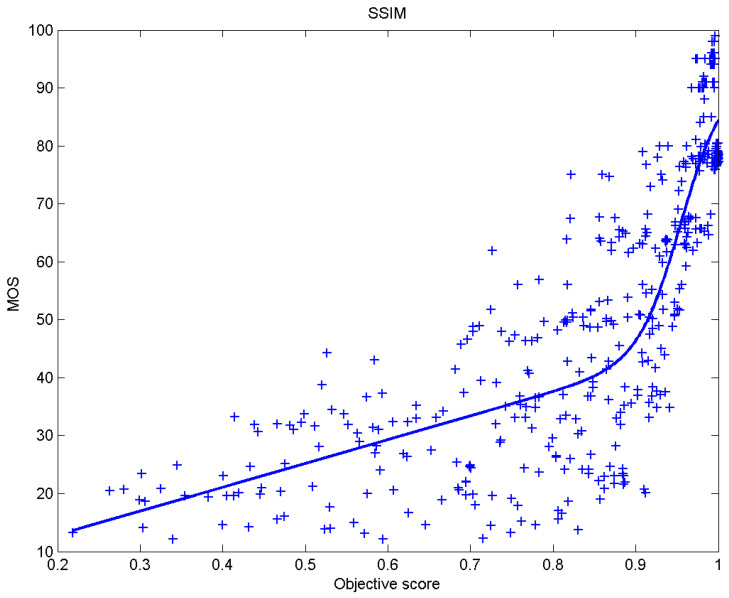
Scatter plots of the subjective scores (MOS) vs. SSIM.

**Figure 20:**
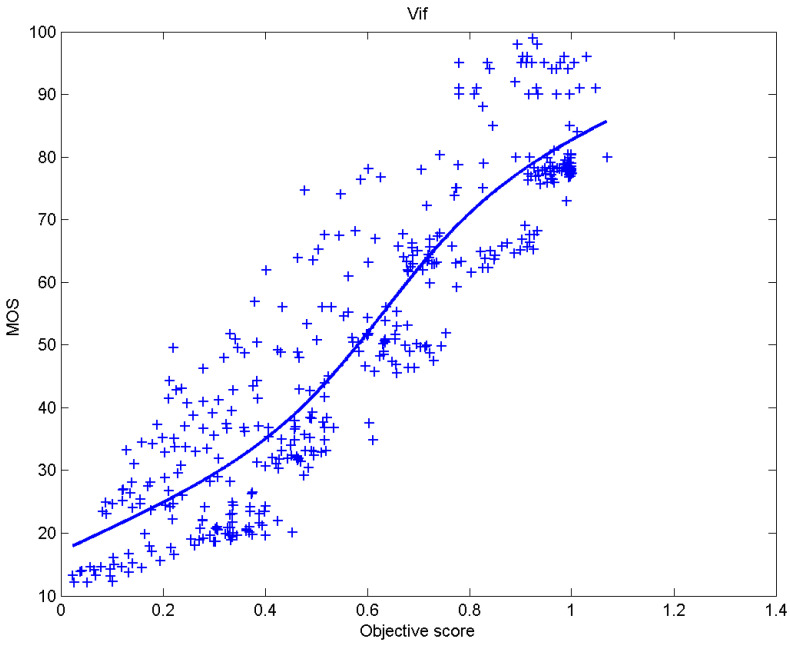
Scatter plots of the subjective scores (MOS) vs. VIF.

**Figure 21:**
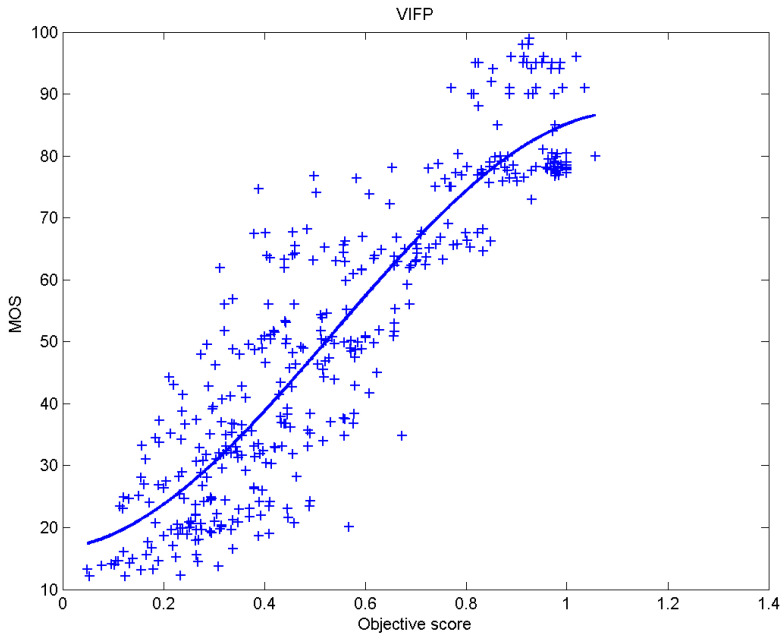
Scatter plots of the subjective scores (MOS) vs. VIFP.

Table 6 presents Pearson’s linear correlation coefficient (PLCC), Spearman’s rank order correlation coefficient (SROCC), Kendall’s rank order correlation coefficient (KROCC) and root–mean–square error (RMSE) evaluated over various image quality metrics (IQM). In order to visualize the results, scatter plots of MOS versus IQM after applying nonlinear regression is presented in Figure 9, Figure 10, Figure 11, Figure 12, Figure 13, Figure 14, Figure 15, Figure 16, Figure 17, Figure 18, Figure 19, Figure 20 and Figure 21.

To evaluate SROCC and KROCC, it is necessary to apply the regression method which provides a non-linear mapping of MOS and the score computed from the IQM [45,55]. The range of correlation coefficients is from 0 to 1. RMSE measure provides the root mean square error between the MOS and objective score after nonlinear regression.

For the nonlinear regression, we used a five-parameter nonlinear monotonic logistic mapping function as given in [44] between the MOS and IQM score.

This can be expressed as $f(x)=\beta_{1}+(\frac{1}{2}-\frac{1}{1+e^{\beta_{2}(x-\beta_{3})}})+\beta_{4}x+\beta_{5}$, where $\beta_{1},\beta_{2},\beta_{3},\beta_{4}\;\mathrm{and}\;\beta_{5}$ are the parameters to be fitted.

## 7. Conclusions

In this paper, the development of NITS-IQA database has been described. Unlike other databases, reference images in the database are captured by the camera in the natural environment, and not taken from photographic CD-ROM such as True Color Kodak Images. In addition to this, analogue images are not used as the reference images in the proposed database. In the NITS-IQA database, some distortions were created using Photoshop which is a unique feature of the database. The subjective experiment of the database was carried out in the laboratory environment. Here, a step by step description of the database development along with the procedure of subjective test experiment was explained. The proposed database is tested over the state-of-the-art IQA metrics for performance evaluation.

## Figures and Tables

**Table 1:** Details of popular image quality databases.

Database Name	Year	Ref. Image	Dist. Image	Dist. Types	Observer	Image Type	Image Format	MOS/ DMOS	MOS/ DMOS Range
MICT [8]	2008	14	168	2	14	Color	bmp	MOS	[1, 5]
IVC [3]	2005	10	185	4	15	Gray	bmp	MOS	[1, 5]
LIVE [6]	2006	29	779	5	161	Color	bmp	DMOS	[0, 100]
A57 [7]	2007	3	54	6	7	Gray	bmp	DMOS	[0, 1]
TID2008 [9]	2008	25	1700	17	838	Color	bmp	MOS	[0, 9]
WIQ [11]	2009	7	80	5	30	Gray	bmp	MOS	[0, 100]
CSIQ [2]	2009	30	866	6	35	Color	png	DMOS	[0, 1]
LAR [10]	2009	8	120	3	20	Color	bmp and ppm	MOS	[1, 5]
TID 2013 [4,5]	2013	25	3000	24	985	Color	bmp	MOS	[0, 9]
KADID-10k [13]	2019	81	10125	25	2209	Color	png	DMOS	[1, 5]
KonIQ-10k [14]	2018	-	10073	-	1467	Color	jpg	MOS	[1, 5]
ChallengeDB [15]	2017	-	1162	-	8100	Color	jpg and bmp	MOS	[0, 100]
SPAQ [16]	2020	-	11125	-	600	Color	jpg	MOS	[0, 100]

**Table 2:** Details of distortions used in popular image quality databases.

Database Name	Developed By	Specific Distortion
MICT [8]	University of Toyama, Japan	(1) JPEG (2) JPEG2000
IVC [3]	University of Nantes, France	(1) JPEG (2) JPEG2000 (3) LAR coding (4) Blurring.
LIVE [6]	University of Texas at Austin	(1) JPEG2000 (2) JPEG (3) White noise (4) Gaussian blur (5) Fast-fading
A57 Database [7]	Image Coding and Analysis Lab, Oklahoma State University	(1) contrast (2) JPEG compression (3) JPEG-2000 compression (4) JPEG-2000+DCQ compression (5) Gaussian blur (6) Gaussian white noise
TID2008 [9]	Tampere Univ. of Tech., Finland	(1) Gaussian noise (2) Additive noise (3) Spatially correlated noise (4) Masked noise (5) High-frequency noise (6) Impulse noise (7) Quantization noise (8) Gaussian blur (9) Image denoising (10) JPEG (11) JPEG2000 (12) JPEG transmission errors (13) JPEG2000 transmission errors (14) Non-eccentricity pattern noise (15) Local block-wise distortions (16) Mean shift (intensity shift) (17) Contrast change
WIQ [11]	Communications and Computer Systems Laboratory, Blekinge Institute of Technology, Sweden	(1) Blocking, (2) Ringing (3) Block intensity shift, (4) Blurring, (5) Noise
CSIQ [2]	Oklahoma State Univ., USA	(1) JPEG (2) JPEG-2000 (3) Gaussian pink noise, (4) Gaussian white noise, (5) Blurring, (6) Contrast
LAR [10]	University of Nantes, France	(1) JPEG (2) JPEG2000 (3) LAR coding
DRIQ [12]	Oklahoma State Univ., USA	Contrast, sharpness, brightness, color, or combination of these properties, with different levels
TID 2013 [4,5]	Tampere Univ. of Tech., Finland	(1) Gaussian noise (2) Additive noise (3) Spatially correlated noise (4) Masked noise (5) High-frequency noise (6) Impulse noise (7) Quantization noise (8) Gaussian blur (9) Image denoising (10) JPEG (11) JPEG2000 (12) JPEG transmission errors (13) JPEG2000 transmission errors (14) Non-eccentricity pattern noise (15) Local block-wise distortions (16) Mean shift (17) Contrast (18) Change of color saturation (19) Multiplicative Gaussian noise (20) Comfort noise (21) Lossy compression of noisy images (22) Image color quantization (23) Chromatic aberrations (24) Sparse sampling and reconstruction

**Table 3:** Summary of descriptive statistical analysis over ten benchmark databases.

Descriptive Statistics	LIVE	CSIQ	A57	MICT	TID	IVC	LAR	WIQ	TID 2013	DRIQ
Mean	0.4034	0.3507	0.3636	0.5340	0.5807	0.5037	0.6316	0.4430	0.6070	0.4345
Standard Error	0.0087	0.0089	0.0371	0.0244	0.0042	0.0231	0.0308	0.0286	0.0032	0.0279
Median	0.4129	0.3232	0.3079	0.5399	0.5917	0.5347	0.7869	0.4264	0.6250	0.4632
Mode	N/A	0.0000	0.0252	0.9356	0.5185	0.8416	0.9899	0.4880	0.5390	N/A
Standard Deviation	0.2420	0.2627	0.2725	0.3411	0.1740	0.3145	0.3375	0.2556	0.1778	0.2468
Variance	0.0586	0.0690	0.0743	0.1163	0.0303	0.0989	0.1139	0.0654	0.0316	0.0609
Kurtosis	−1.0516	−0.8453	−0.5148	−1.5216	0.1420	−1.4043	−1.3188	−0.4470	−0.1416	−1.0680
Skewness	0.1198	0.4664	0.6246	−0.1206	−0.4932	−0.1331	−0.4948	0.4443	−0.5549	0.0617
No. of images	779	866	54	196	1700	185	120	80	3000	78
Confidence Level (95.0%)	0.0170	0.0175	0.0744	0.0480	0.0083	0.0456	0.0610	0.0569	0.0064	0.0556
Coefficient of Variation in %	59.99	74.91	74.94	63.88	29.96	62.44	53.44	57.70	29.29	56.80

**Table 4:** Details of the used distortions in NITS-IQA database.

Method	Purpose	Occurred during	Parameters	Low Level	High Level
Gaussian Blur	Smoothen image	Image Acquisition	Filter radius	0.1	1000
Gaussian Noise	Illumination can be poor or temperature can be high	Image Acquisition and Transmission	Filter	0.1	400
Uniform Noise (Quantization noise)	Quantization of the pixel	Image Acquisition Image registration			
	Filter	0.1	400		
Contrast change (Without Brightness change)	Adjust the contrast	Image acquisition, gamma correction	Contrast gain	−50	100
Pixelate Mosaic	Pixelation	Image registration	Filter	2	200
Motion Blur	Capturing at single exposure, either due to movement or long exposure	Image acquisition	Filter radius	1	2000
JPEG	Compromise between image size and its quality	JPEG Compression, image acquisition, image storage, image transmission	Encoding	1	100
JPEG2000	Compression	JPEG2000 Compression, image acquisition, image storage, image transmission	Encoding	1	100
JPEG-XT	Compression	JPEG-XT Compression, image acquisition, image storage, image transmission	Encoding	1	100

**Table 5:** Compilation of distortions in the NITS-IQA database in terms of range and level of distortion.

Distortion	Range	Unit	Levels 1	Levels 2	Levels 3	Levels 4	Levels 5
Gaussian Blur	0.1–1000	pixels	0.2	0.9	1.5	3	6
Gaussian Noise (Chromatic)	0.1–400%	percentage	1	2	4	7	11
Uniform Noise (Chromatic)	0.1–400%	percentage	1	2	5	11	17
Contrast change (Without Brightness change)	−150	points	−50	−25	25	50	100
Pixelate Mosaic	2 to 200	cells	2	3	4	5	6
Motion Blur	1 to 2000	pixels	1	2	4	6	10
JPEG	1–100	bpp	12	20	35	50	99
JPEG2000	1–100	bpp	1	0.03	0.01	0.07	0.05
JPEG-XT	1–100	bpp	12	20	35	50	99

**Table 6:** Pearson’s linear correlation coefficient (PLCC), Spearman’s rank order correlation coefficient (SROCC), Kendall’s rank order correlation coefficient (KROCC) and root–mean–square error (RMSE) for the various image quality metrics (IQM).

IQM	PLCC	SROCC	KROCC	RMSE
SSIM [38]	0.8659	0.8489	0.6434	12.0473
IFC [39]	0.8878	0.8802	0.6858	11.0865
VIFP [40]	0.8976	0.8858	0.6962	10.6189
VSNR [41]	0.5903	0.6608	0.4991	19.4409
P_HVS_M [42]	0.5392	0.5923	0.4727	20.2838
P_HVS [43]	0.6241	0.6363	0.4923	18.8186
RFSIM [44]	0.8178	0.8171	0.6145	13.8616
FSIM [45]	0.8807	0.8774	0.6863	11.4104
ADM [46]	0.8796	0.8780	0.6918	11.4592
IWSSIM [47]	0.8799	0.8787	0.6884	11.4451
IWMSE [47]	0.5493	0.5994	0.4980	20.1258
IWPSNR [47]	0.5896	0.5994	0.4979	19.4532
SRSIM [48]	0.6566	0.8543	0.6614	18.1656
GSM [49]	0.8593	0.8577	0.6686	12.3172
IGM [50]	0.8656	0.8633	0.6755	12.0617
GMSD [51]	0.8918	0.8893	0.7008	10.8958
MSE	0.6607	0.6745	0.4980	18.0792
PSNR	0.6512	0.6638	0.4915	18.2776
UQI [52]	0.8841	0.8744	0.6770	11.2542
MSSIM [53]	0.8690	0.8691	0.6724	11.9163
WSNR [47]	0.8878	0.8802	0.6858	11.0865
SGF [54]	0.8984	0.889	0.697	10.579979

## Data Availability

The NITS-IQA database is available at: https://drive.google.com/drive/folders/0B_bnn8Xh3PMmT1VxSlVRWDNCTk0?resourcekey=0-9JzjQxVUNJXIodLwkiZ-Lg&usp=sharing (accessed on 18 January 2023).
